# Mediating factors on the association between fear of falling and health-related quality of life in community-dwelling German older people: a cross-sectional study

**DOI:** 10.1186/s12877-020-01802-6

**Published:** 2020-10-14

**Authors:** Sophie Gottschalk, Hans-Helmut König, Michael Schwenk, Carl-Philipp Jansen, Corinna Nerz, Clemens Becker, Jochen Klenk, Judith Dams

**Affiliations:** 1 Department of Health Economics and Health Services Research, University Medical Center Hamburg-Eppendorf, Hamburg Center for Health Economics, Martinistraße 52, 20246 Hamburg, Germany; 2 Network Aging Research, Heidelberg University, Heidelberg, Germany; 3 Department of Clinical Gerontology and Geriatric Rehabilitation, Robert Bosch Hospital, Stuttgart, Germany; 4 Institute of Epidemiology and Medical Biometry, Ulm University, Ulm, Germany; 5 IB University of Applied Health and Social Sciences, Study Centre Stuttgart, Stuttgart, Germany

**Keywords:** Fear of falling, EQ-5D, Health-related quality of life, Falls efficacy, Older persons

## Abstract

**Background:**

Previous research has shown that not only falls, but also fear of falling (FoF) influences health-related quality of life (HrQoL) negatively. The EQ-5D (consisting of an index and a visual analogue scale [EQ-VAS]) is a frequently used instrument to determine HrQoL in clinical studies and economic evaluations, but no previous study compared the association between FoF and the EQ-5D index with the association between FoF and the EQ-VAS. Moreover, factors that influence the association between FoF and HrQoL are rarely examined. Thus, this study aimed to examine the association between FoF and HrQoL and to examine factors that mediate the association.

**Methods:**

FoF (Short Falls Efficacy Scale International) and HrQoL (EQ-5D descriptive system, EQ-5D index, and EQ-VAS) were assessed in a sample of community-dwelling older persons (≥70 years) participating in the baseline assessment of a randomized controlled trial (*N* = 309). Linear and logistic regression analyses were performed, adjusting for sociodemographic variables, frequency of falls, number of chronic conditions, functional mobility (Timed up-and-go test), and subjective functional capacity (LLFDI function and disability scales). Multiple regression models were used to test the mediating effects.

**Results:**

Moderate or high FoF was prevalent in 66% of the sample. After adjusting for covariates, FoF was negatively associated with the EQ-5D index, but not with the descriptive system or the EQ-VAS. Subjective functional capacity partly mediated the association between FoF and the EQ-5D index and completely mediated the association between FoF and the EQ-VAS.

**Conclusion:**

FoF was negatively associated with the EQ-5D index. As subjective functional capacity mediated the association between FoF and HrQoL, future interventions should account for subjective functional capacity in their design.

## Background

Due to the demographic change, the older population is projected to increase [1]. As this population typically has a higher level of (multi-)morbidity, an increase will probably pose challenges to health care systems in the future. The reasons for higher morbidity in older age are manifold. However, especially falls are a frequent health-deteriorating event in older people. One third of the population aged 65 years and above experiences a fall within a year [2–6], which often leads to severe consequences like injuries, or activity limitations, and consequently, to a decline in health-related quality of life (HrQoL) [7–9].

Since many health systems move beyond the idea of mere survival but focus on maintaining the best possible health status, overarching concepts like HrQoL have become more important in describing the impact of health conditions or the effects of interventions. HrQoL is subjective and depends on a variety of physical, emotional, and social-cultural factors [10, 11]. It is therefore necessary to take the individual valuation of the health status into account. Several measurements of HrQoL have been developed. The EQ-5D [12, 13] is a generic instrument which is widely used in clinical studies and economic evaluations. It comprises a descriptive system and a visual analogue scale (EQ-VAS). The descriptive system of the EQ-5D can be transformed to an index based on societal preference values, whereas the EQ-VAS quantifies the overall current health status based on a respondent’s individual preferences [14].

As a recent systematic review by Schoene et al. [15] confirmed, not only falls but also fall-related risk factors like fear of falling (FoF) influence HrQoL negatively. In the past, FoF was considered as consequence of falls, but nowadays FoF is considered as an independent predictor of disability or HrQoL, independent of a prior fall experience [15, 16]. The prevalence of FoF in the population aged 65 years and above varies widely, with the majority of studies reporting a prevalence between 20 and 85% depending on sample characteristics and the measurement used to assess FoF [15, 17]. The prevalence of FoF tends to be higher in females, older persons, as well as in those having a history of falls, being physically impaired, or reporting poor self-rated health [17–19]. Moreover, psychological factors, such as depressive symptoms, loneliness, optimism, or self-esteem, are related to FoF [20, 21]. Consequences of FoF are a decline in cognitive and physical function, higher physical dependence, an increased risk of falling, the avoidance of activities, and restrictions in participation in social activities [17, 22–30].

The review concluded that the association between FoF and HrQoL was consistent, regardless of the instruments used to assess FoF and HrQoL, with the majority of studies using generic multidimensional instruments of HrQoL, like the EQ-5D or the SF-36, and validated instruments of FoF [15]. But these studies on the association between FoF and HrQoL mainly examined FoF as independent predictor [31–34], whereas the factors influencing the association between FoF and HrQoL were hardly addressed. However, identifying these factors is crucial as they might be modifiable [35] and could therefore be considered in the development of interventions. As the risks and consequences of FoF themselves predict HrQoL [33, 36, 37], it is reasonable to assume that they mediate the association between FoF and HrQoL. To our knowledge, only one study explored mediating effects. Using samples of community-dwelling older persons from Germany (*n* = 182) and Taiwan (*n* = 193), Hsu et al. [38] found that the association between FOF and HrQoL, measured using the SF-12, was significantly mediated by the self-concept of health and physical activity.

The EQ-5D is the most frequently used instrument to determine HrQoL in clinical studies and economic evaluations, but no previous study on the association between FoF and HrQoL compared the association between FoF and the EQ-5D index with the association between FoF and the EQ-VAS [15]. Therefore, the current study aimed to close this gap. In addition, the current study focused on factors that mediate the association between FoF and HrQoL in order to better understand the mechanisms underlying this association, which may serve as a basis for new approaches in the design of interventions.

## Methods

### Sample description/characteristics

Baseline data was taken from a multi-centre, two armed, single-blinded, randomized fall prevention trial (LiFE-is-LiFE) evaluating a group-based version of the ‘Lifestyle-integrated Functional Exercise’ Program (LiFE) [39] for its non-inferiority compared to the original face-to-face approach [40].

The LiFE-is-LiFE trial included community-dwelling, German-speaking people aged ≥70 years with a history or risk of falling (> 2 falls or 1 injurious fall within the last 12 month or limited balance [Timed Up-and-Go time ≥ 12 s]), who were able to ambulate 200 m without personal assistance. Participants were excluded if they exceeded a certain physical activity level (structured exercise > 1 time per week or self-reported activity level above 150 min of moderate to vigorous physical activity per week in past 3 months), were unavailable for home visits during the intervention time or for completion of the follow-up assessments, if they participated in another scientific trial, or had certain medical conditions that affect the ability to perform the activities taught in the program (e.g., Parkinson’s disease or moderate to severe cognitive impairment). A detailed description of the LiFE-is-LiFE project and its inclusion and exclusion criteria can be found elsewhere [40].

### Health-related quality of life

HrQoL was assessed using the EQ-5D-5L questionnaire [12, 41]. The EQ-5D-5L descriptive system comprises the five dimensions mobility, self-care, usual activities, pain/discomfort and anxiety/depression. In each dimension, study participants were asked to rate their health problems on an ordinal five level scale with “no problems (1)”, “slight problems (2)”, “moderate problems (3)”, “severe problems (4)” or “extreme problems (5)”. By combining the answers, an individual health state out of 3125 (5^5^) possible health states was obtained for each participant, with “11111” and “55555” representing the best and worst health state, respectively. Health states were transformed to an index value based on preference-based value sets from the German general population [42]. Since there are health states of the reference population being predicted to be < 0 [42], the EQ-5D index can take values between − 0.662 representing the worst possible HrQoL, 0 representing death, and 1 representing the best possible HrQoL. Generally, a value < 0 is assumed to present a health state which is valued worse than death.

In addition to the descriptive system and the EQ-5D index, HrQoL was assessed on a visual analogue scale (EQ-VAS). Participants were asked to rate their overall current health between 0 (worst) and 100 (best) [12].

### Fear of falling

FoF was assessed with the German version of the Short Falls Efficacy Scale International (Short FES-I) [43]. Participants were asked to rate their concerns about falling regarding the execution of seven everyday tasks on a 4-level Likert scale reaching from “not at all concerned” (1), “somewhat concerned” (2), “fairly concerned” (3), to “very concerned” (4). A Short FES-I sum score was calculated by adding up the answers. This score ranged from 7 (“no concern about falling”) to 28 (“severe concern about falling”) with low, moderate and high concern represented by a score between 7 and 8, 9–13, and 14–28, respectively [44].

### Further measurements

The frequency of falls was assessed by the self-reported number of injurious or non-injurious falls in the previous 6 months.

The number of chronic conditions was assessed by a sum score of the following chronic conditions: diabetes type 1 and 2, hypertension, acute cardiovascular disease, a history of heart attacks, a cardiac defect, auricular fibrillation or other cardiac arrhythmias, a history of stroke (more than 6 month ago) or transient ischemic attacks, arthrosis, rheumatoid arthritis, cancer (not on active treatment), asthma or chronic obstructive pulmonary disease (Gold class < III), osteoporosis, or depression.

Functional mobility was assessed via the Timed Up-and-Go Test (TUG) measuring the time a person needs to get up from a chair, walk three meters at a comfortable and safe pace, return, and sit down again [45].

Subjective functional capacity was measured using the Late Life Function and Disability Instrument (LLFDI) [46, 47], an instrument designed to assess physical functioning in older adults based on a theoretical or conceptual model that characterizes physical functioning within a socio-medical model of disability. It measures two distinct outcomes: function and disability. In the 32-item LLFDI function component, participants rate their ability to perform discrete actions or activities on a 5-level Likert scale (“no”, “slight”, “moderate”, “heavy”, or “total limitations”). In the 16-item LLFDI disability component, the participants’ limitations in performing specific life tasks within a typical sociocultural and physical environment are assessed on a 5-level Likert scale (“not at all”, “a little”, “somewhat”, “a lot”, or “completely”). In the current study, the second LLFDI disability dimension focusing on frequency of performance was skipped. For both components (function and disability), a sum score was calculated and transformed to a scale between 0 and 100, with lower scores indicating a higher level of functional limitations or disability.

Sociodemographic variables comprised age, sex, educational status, marital status (married or living in a partnership/widowed/divorced/permanently living separated/single) and living situation (living alone/living with others). Educational status was measured by the highest school leaving qualification achieved. Since the information was assessed based on qualification levels, which are specific for the German educational system, the information was grouped into “low” (9 years of school education), “intermediate” (10 years of school education), and “high” (qualifies to enter university) level of education.

### Statistical analysis

In addition to descriptive statistics, the association between FoF and HrQoL measured using the EQ-5D index, the EQ-5D descriptive system and the EQ-VAS was examined using Spearman’s rank correlation coefficients. According to Cohen, correlation coefficients between 0.10–0.19, 0.30–0.49 and 0.50–1.00 were interpreted as weak, moderate, and strong, respectively [48]. Furthermore, linear regression models were performed with the EQ-5D index or the EQ-VAS as dependent variables and FoF as independent variable. Neither the EQ-5D index nor the EQ-VAS was distributed normally, thus bootstrapped standard errors and confidence intervals for the regression coefficients from 10,000 resampled data sets were estimated. To examine the association between FoF and the EQ-5D descriptive system, logistic regression models were performed by dichotomizing answers of each EQ-5D dimension, with 0 representing no problems and 1 representing any problems. For each outcome, two models were calculated: the first model (Model 1) included FoF and sociodemographic variables, whereas the second model (Model 2) additionally included the number of chronic conditions, the number of falls, functional mobility (TUG), and subjective functional capacity (LLFDI function and disability scales). Additionally, path models were performed to estimate the mediating effects of function and disability on the association between FoF and EQ-5D-index and EQ-VAS following the Baron and Kenny approach [49]. The indirect effects were tested for significance using the Sobel test [50].

Statistical analyses were conducted using STATA/SE 16.0 [StataCorp. 2019. Stata Statistical Software: Release 16. College Station, TX: StataCorp LLC]. For all analyses, the significance level was set to 0.05.

## Results

### Descriptive statistics

Sociodemographic and clinical characteristics are presented in Table 1. At baseline *N* = 309 participants were included with a mean age of 78.68 (standard deviation [SD] 5.31) years. The majority was female (73.46%) and had an intermediate or high educational level (67.96%). Approximately half of the sample was married or was living in a partnership (45.31%), whereas one third was widowed (35.92%) and one tenth was divorced (11.97%). Only a small proportion was single (5.83%) or permanently living separated (0.97%). On average, participants reported 2.52 (SD 1.56) chronic conditions. The mean scores on the LLDFI function and disability scales were 57.34 (SD 7.94) and 70.66 (SD 11.98), respectively. In the previous 6 months, 40.78% of the sample experienced at least one fall. Among those who fell, the average number of falls was 1.61 (SD 1.21). Low FoF was reported by 33.98%, whereas 52.75% reported moderate FOF and 13.27% reported high FoF. The mean EQ-5D index was 0.84 (SD 0.15) and the mean EQ-VAS was 70.91 (SD 16.46). Furthermore, differences between people experiencing at least one fall and those without falls were not significant (data not shown).

**Table 1:** Sample characteristics

*N* = 309			
**Female**	n (%)	227	73.46
**Age**	Mean (SD)	78.67	5.31
**Educational status**	n (%)		
Low		94	30.42
Intermediate		92	29.77
High		118	38.19
Other		5	1.62
**Marital status**	n (%)		
Married/living in a partnership		140	45.31
Widowed		111	35.92
Divorced		37	11.97
Permanently living separated		3	0.97
Single		18	5.83
**Living alone**	n (%)	166	53.72
**Chronic conditions**	Mean (SD)	2.52	1.56
**LLFDI function**^**a**^	Mean (SD)	57.34	7.94
**LLFDI disability**^**b**^	Mean (SD)	70.66	11.98
**TUG** (time in seconds)	Mean (SD)	13.29	3.86
**Prevalence of fallers**	n (%)	126	40.78
**Number of falls among fallers**	Mean (SD)	1.61	1.21
**Fear of falling**	n (%)	10.36	3.03
Low concern		105	33.98
Moderate concern		163	52.75
High concern		41	13.27
**EQ-5D index**	Mean (SD)	0.84	0.15
**EQ-VAS**	Mean (SD)	70.91	16.46

*LLFDI* Late Life Function and Disability Instrument, *TUG* Timed up-and-go test

^a^Higher score indicates lower limitations

^b^Higher score indicates lower disability

### Correlation coefficients

In bivariate analyses (Table 2), Spearman’s rank correlations between FoF and the EQ-5D index, EQ-5D descriptive system or EQ-VAS were weak to moderate, with absolute correlation coefficients between r_S_ = 0.17 (*p* < 0.05) and 0.43 (*p* < 0.05). Furthermore, associations between FoF and the living situation and functional mobility were weak (r_S_ = 0.15 and 0.24, *p* < 0.05). Moreover, FoF correlated strongly with function (r_S_ = 0.56, *p* < 0.05) and moderately with disability (r_S_ = 0.43, *p* < 0.05). No significant correlation was found between FoF and age, gender, educational level, the number of chronic conditions, or the number of falls (*p* > 0.05).

**Table 2:** Correlation coefficients between fear of falling and variables of health, functional status, and sociodemographic characteristics

Variables	FES-I
EQ-5D index	−0.43*
EQ mobility	0.29*
EQ self-care	0.35*
EQ usual activities	0.34*
EQ pain/discomfort	0.17*
EQ anxiety/depression	0.25*
EQ VAS	−0.28*

*p < 0.05

### Multivariate regressions

#### Association between FoF and sub-dimensions of HrQoL (EQ-5D descriptive system)

Results of the logistic regression models for the association between FoF and the dimensions of the EQ-5D descriptive system are presented in Table 3. After adjusting for sociodemographic variables (Model 1), FoF was significantly associated with problems in each dimension (odds ratios [OR] between 1.14 and 1.35, *p* < 0.05). These associations became non-significant after adjusting for chronic conditions, functional mobility, and subjective functional capacity (Model 2).

**Table 3:** Logistic regression models of the association between fear of falling and the EQ-5D descriptive system

*N* = 309	EQ-5D Dimensions									
	Mobility		Self-care		Usual activities		Pain/Discomfort		Anxiety/Depression	
	Model 1	Model 2	Model 1	Model 2	Model 1	Model 2	Model 1	Model 2	Model 1	Model 2
Fear of falling	1.30*** (1.17–1.45)	1.05 (0.93–1.20)	1.35*** (1.22–1.50)	1.12 (0.99–1.26)	1.35*** (1.22–1.50)	1.12 (0.99–1.26)	1.14* (1.03–1.27)	0.93 (0.81–1.06)	1.18*** (1.08–1.28)	1.11 (1.00–1.23)
Age	1.00 (0.96–1.05)	10.97 (0.92–1.02)	1.02 (0.97–1.07)	0.99 (0.93–1.04)	1.02 (0.97–1.07)	0.99 (0.93–1.04)	0.96 (0.91–1.00)	0.93* (0.88–0.98)	0.95 (0.91–1.00)	0.92** (0.87–0.97)
Female	0.82 (0.45–1.48)	0.52 (0.26–1.03)	0.98 (0.55–1.76)	0.69 (0.35–1.34)	0.98 (0.55–1.76)	0.69 (0.35–1.34)	1.40 (0.74–2.67)	0.90 (0.45–1.82)	1.31 (0.71–2.42)	1.39 (0.72–2.69)
Education										
High	ref.	ref.	ref.	ref.	ref.	ref.	ref.	ref.	ref.	ref.
Low	1.86* (1.01–3.40)	1.55 (0.79–3.04)	1.39 (0.77–2.52)	1.20 (0.62–2.33)	1.39 (0.77–2.52)	1.20 (0.62–2.33)	2.64** (1.33–5.26)	2.36* (1.13–4.94)	1.92* (1.05–3.53)	2.09* (1.10–4.00)
Intermediate	1.31 (0.72–2.38)	1.08 (0.57–2.06)	1.53 (0.84–2.78)	1.20 (0.63–2.31)	1.53 (0.84–2.78)	1.20 (0.63–2.31)	1.49 (0.79–2.81)	1.39 (0.70–2.73)	1.83 (0.99–3.38)	1.47 (0.78–2.79)
Other	0.73 (0.10–5.39)	0.68 (0.05–8.79)	3.58 (0.33–39.0)	6.58 (0.37–116)	3.58 (0.33–39.0)	6.58 (0.37–116)	1.94 (0.19–19.9)	2.34 (0.18–31.0)	3.61 (0.52–24.9)	2.90 (0.34–24.9)
Shared living	0.75 (0.44–1.26)	0.88 (0.50–1.56)	0.95 (0.57–1.61)	1.13 (0.64–2.01)	0.95 (0.57–1.61)	1.13 (0.64–2.01)	1.69 (0.93–3.04)	2.38** (1.24–4.55)	0.92 (0.54–1.56)	0.93 (0.53–1.62)
Chronic conditions		1.15 (0.96–1.40)		0.95 (0.80–1.14)		0.95 (0.80–1.14)		1.02 (0.83–1.25)		0.99 (0.83–1.17)
Number of falls		1.09 (0.82–1.44)		1.07 (0.82–1.39)		1.07 (0.82–1.39)		0.96 (0.71–1.30)		0.99 (0.78–1.25)
Function		0.89*** (0.84–0.95)		0.92** (0.87–0.97)		0.92** (0.87–0.97)		0.87*** (0.82–0.93)		1.03 (0.98–1.08)
Disability		0.97* (0.94–0.99)		0.94*** (0.92–0.97)		0.94*** (0.92–0.97)		0.99 (0.96–1.02)		0.95*** (0.92–0.98)
Functional mobility		1.02 (0.93–1.13)		1.02 (0.93–1.12)		1.02 (0.93–1.12)		0.98 (0.90–1.08)		1.09* (1.00–1.19)

Odds ratios with 95% CI in parentheses; *** *p* < 0.001, ** *p* < 0.01, * *p* < 0.05; fear of falling was assessed with the Short Falls-efficacy Scale-International (Short-FES-I), function and disability with the Late-life Function and Disability Instrument (LLFDI), and functional mobility with the Timed up-and-go test

#### Association between FoF and EQ-5D index

After adjusting for sociodemographic variables (Model 1), linear regression revealed a significant negative association between FoF and the EQ-5D index (β = − 0.02, *p* < 0.001; Table 4). This relationship remained significant after adjusting for chronic conditions, functional mobility, and subjective functional capacity (ß = -0.01, *p* < 0.01; Model 2).

**Table 4:** Linear regression models of the association between fear of falling and EQ-5D-rated and EQ-VAS-rated HrQoL

*N* = 309	EQ-5D Index				EQ VAS			
	Model 1		Model 2		Model 1		Model 2	
	β	SE	β	SE	β	SE	β	SE
Fear of falling	**−0.023** ***	(0.004)	**−0.010** **	(0.004)	**−1.535** ***	(0.354)	−0.361	(0.396)
Age	0.001	(0.001)	**0.004** *	(0.001)	0.049	(0.169)	0.228	(0.162)
Female	−0.014	(0.016)	0.007	(0.014)	−0.370	(2.169)	1.527	(2.135)
Education								
High	ref.		ref.		ref.		ref.	
Low	**−0.050** **	(0.018)	−0.033	(0.017)	−2.342	(2.128)	−0.571	(2.018)
Intermediate	**−0.039** *	(0.018)	−0.022	(0.016)	− 2.126	(2.235)	−0.403	(2.209)
Other	−0.036	(0.025)	−0.027	(0.053)	−0.471	(5.543)	0.055	(3.549)
Shared living	−0.002	(0.016)	−0.013	(0.014)	1.511	(1.893)	0.575	(1.779)
Chronic conditions			**−0.014** **	(0.005)			**−1.761** **	(0.573)
Number of falls			0.002	(0.006)			0.370	(0.974)
Function			**0.005** ***	(0.001)			**0.533** ***	(0.154)
Disability			**0.002** **	(0.001)			**0.208** **	(0.080)
Functional mobility			−0.003	(0.003)			0.065	(0.297)
Adj. R-Squared	0.233		0.379		0.072		0.188	

Fear of falling was assessed with the Short Falls-efficacy Scale-International (Short-FES-I), function and disability with the Late-life Function and Disability Instrument (LLFDI), and functional mobility with the Timed up-and-go test

*** *p* < 0.001, ** *p* < 0.01, * *p* < 0.05

#### Association between FoF and EQ-VAS

In Model 1, higher FoF was significantly associated with a lower EQ-VAS score (β = − 1.54, *p* < 0.001; Table 4). After adjusting for chronic conditions, functional mobility, and subjective functional capacity (Model 2), FoF did no longer significantly predict the EQ-VAS score (β = − 0.36, *p* > 0.05), whereas the number of comorbidities (β = − 1.76, *p* < 0.01), and the levels of function (β = 0.53, *p* < 0.001) and disability (β = 0.21, *p* < 0.01) significantly predicted the EQ-VAS.

### Mediating effects of function and disability

**Fig. 1:**
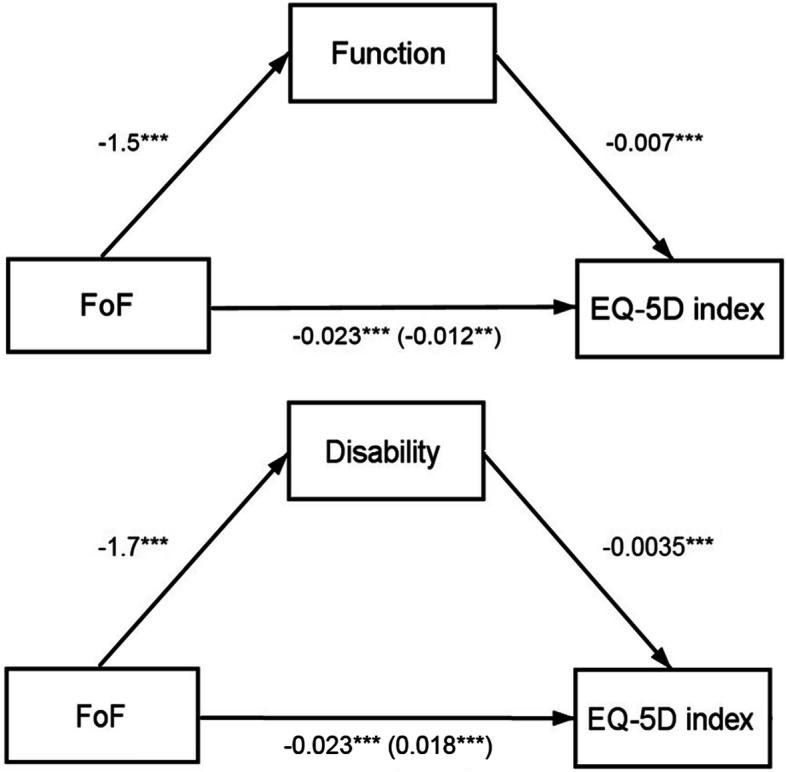
Mediating effects of function and disability on the association between FoF and HrQoL (EQ-5D index). Note: Path diagrams indicate that function and disability partially mediated the association between fear of falling (FoF) and EQ-5D-rated health-related quality of life. Numbers outside the parentheses denote the path coefficients between variables, whereas numbers in the parentheses indicate the path coefficients after including the mediator (direct effect). Function and disability were assessed with the Late-Life Function and Disability Instrument (LLFDI). *p < 0.05, **p < 0.01, ***p < 0.001

**Fig. 2:**
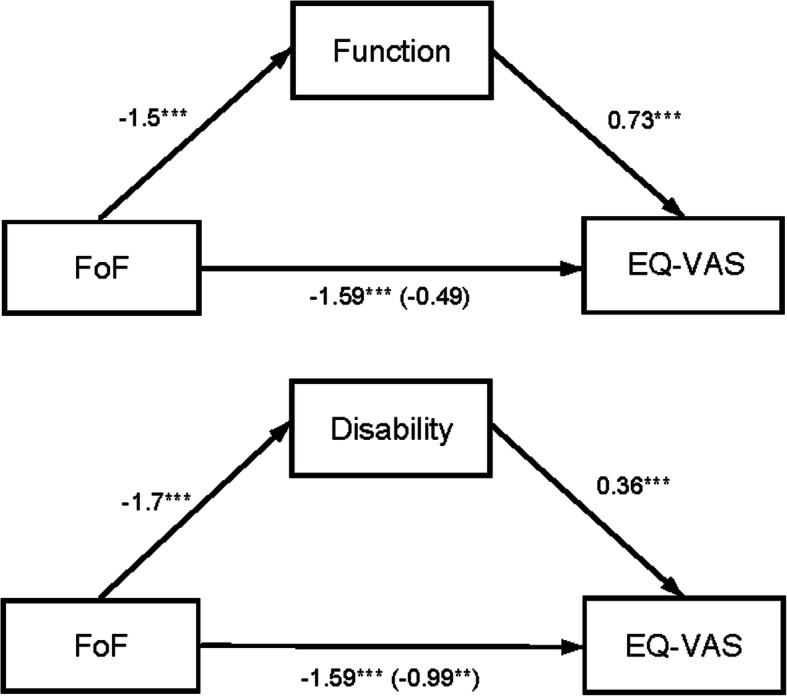
Mediating effects of function and disability on the association between FoF and HrQoL (EQ-VAS). Note: Path diagrams indicate that function completely mediated and disability partially mediated the association between fear of falling (FoF) and EQ-VAS-rated health-related quality of life. Numbers outside the parentheses denote the path coefficients between variables, whereas numbers in the parentheses indicate the path coefficients after including the mediator (direct effect). Function and disability were assessed with the Late-Life Function and Disability Instrument (LLFDI). *p < 0.05, **p < 0.01, ***p < 0.001

Figures 1 and 2 show the mediation results of self-reported function and disability on the relationship between FoF and HrQoL. Separate mediation models were calculated for function and disability. Function and disability partially mediated the association between FoF and the EQ-5D index. The coefficient of FoF increased from − 0.023 to − 0.012 after controlling for function (Sobel test Z = − 3.08, *p* < 0.01) and to − 0.018 after controlling for disability (Sobel test Z = − 5.31, *p* < 0.001). The association between FoF and the EQ-VAS was completely mediated by function as the coefficient of FoF increased from − 1.589 to a non-significant effect of − 0.489 (Sobel test Z = − 1.25, *p* > 0.05) after controlling for function. After controlling for disability, the coefficient of FoF increased from − 1.589 to − 0.989 (Sobel test Z = -2.69, *p* < 0.01), indicating a partial mediating effect of disability on the association between FoF and the EQ-VAS.

## Discussion

In this sample of community-dwelling older persons from Germany, 66% had moderate or high FoF. After adjustment for sociodemographic characteristics, chronic conditions, functional mobility, and subjective functional capacity, FoF was significantly associated with HrQoL measured by the EQ-5D index. This confirmed previous findings [15]. The current study did not only examine the influence of FoF on the EQ-5D index or on the EQ-5D descriptive system, but also the influence of FoF on the overall current health status (EQ-VAS). When accounting for sociodemographic characteristics, chronic conditions, functional mobility, and subjective functional capacity, FoF was not associated with the EQ-VAS. Overall, FoF seemed to be better captured by the specific EQ-5D dimensions than by the unspecific assessment of the EQ-VAS. This may be due to the different concepts underlying the EQ-5D index and the EQ-VAS. The EQ-5D index is based on subjective evaluations of health in five specific dimensions. However, these subjective ratings in the respective dimensions were transformed into an index based on societal preference values. These values were obtained using a representative sample of the general population and thus reflect the societal weighting of restrictions in the respective dimensions of the EQ-5D descriptive system [42]. In contrast, the EQ-VAS is subject to a valuation of health based on individual preferences. Thus, by asking how healthy participants felt today on the EQ-VAS without giving predefined dimensions like in the EQ-5D descriptive system, aspects other than FoF may play a greater role for participants in assessing their overall current health.

As already found in another study [31], higher age was associated with better EQ-5D-rated HrQoL. This can probably be explained by a selection bias. When the distribution of HrQoL and age were visually assessed, participants aged 87 and older exclusively reported EQ-5D index values above 0.7, whereas in younger participants, the EQ-5D index values of some individuals were also distributed at lower levels. When excluding participants aged 87 and older in additional analyses, age was no longer significantly associated with HrQoL.

Contrary to previous studies, where falls and FoF were associated and both had a significant relationship with HrQoL [15, 34], no significant association between the number of previous falls and HrQoL was found in the current study. However, the information on the history of falls was based solely on retrospective self-reports and may therefore be biased. Instead, factors like chronic conditions and activity restrictions (function and disability) seemed to be more important than falls in the association between FoF and HrQoL. Mediation analyses showed that function and disability partially mediated the association between FoF and the EQ-5D index. With regard to the EQ-VAS, the effect of FoF was partially mediated by disability and completely mediated by function. These findings are not surprising as previous research suggested that FoF is linked to disability and deteriorating function [51, 52]. Moreover, the FES-I measures FoF in the context of typical everyday activities. It therefore seems obvious that functional limitations and disability are reflected in FoF. Although the current study was of cross-sectional nature and therefore no causal inferences can be drawn from the results of the mediation analyses, possible interpretations of these findings can be hypothesized. A certain degree of FoF may protect individuals from an actual fall, because they are more attentive or careful [53]. However, when FoF leads to the avoidance of certain activities, it becomes a vicious circle. Avoiding activities leads to a deterioration in physical functioning, which in turn leads to an increased risk and fear of falling [2, 23, 25, 27, 28, 54–56]. Actual falls again lead to a further deterioration in physical health status [9, 30, 57]. This reduction in physical health status and social activities ultimately results in a higher level of dependence and poorer HrQoL [9, 30, 58, 59]. That function completely mediated the association between FoF and the EQ-VAS in this study may indicate that limitations in doing discrete actions or activities (function) play a greater role in the evaluation of overall current health (EQ-VAS) than the capability of performing less discrete, socially defined life tasks (disability). Furthermore, it may reflect a strong link between FoF and functional limitations. High FoF may prevent people from doing certain activities but may not prevent them from finding solutions to adapt to their FoF and functional limitations which enables them to perform socially defined life tasks despite FoF.

The mediating effect of function and disability in the current study emphasizes the importance to maintain daily and social life activities in older people. Thus, addressing these factors in interventions may lead to a reduction of FoF and an improvement in HrQoL. A randomized controlled trial from the Netherlands evaluated a home-based cognitive behavioural program to encourage older persons in performing activities of daily living [60]. The intervention focused on the identification and restructuring of misconceptions about falls, as well as on the uptake of new or previously avoided daily life activities and their safe execution. Thereby, the home-based cognitive behavioural program was effective in reducing FoF and disability. However, since the effect sizes of FoF, function, and disability on HrQoL were rather small in the current study, considering these factors alone in interventions may not lead to clinically important changes in HrQoL, which is related to the multidimensionality of factors influencing HrQoL.

### Strengths and limitations

Even though different studies have already reached consensus regarding the association between FoF and HrQoL [15], to our knowledge, no previous study compared the association between FoF and the EQ-5D index (a multidimensional measure of HrQoL) with the association between FoF and the EQ-VAS (a single-item measure of overall current health). Moreover, previous findings were extended by examining the mediating effects of function and disability on the association between FoF and HrQoL.

This study has some limitations**.** As the current study used cross-sectional data, mediation models may not reflect the true direction of influence. Instead of being a consequence of FoF, functional limitations may lead to FoF, or even both directions of influence exist. Due to the conceptual overlap between function and disability, separate mediation models for function and disability were calculated, which precludes investigating their independent contributions. However, the mediation results of this study may serve as basis for future studies, which could, for example, investigate the causal relationship between FoF and HrQoL more closely using longitudinal data or by calculating more complex path models. In addition, the sample size of *N* = 309 was relatively small, thus results may not be generalizable to the older population at risk of falling in Germany. Furthermore, the selected sample reached better EQ-5D index values compared with normative values for the general German population of the respective age group [61]. This is most likely due to the exclusion of individuals who were cognitively impaired and had certain chronic conditions. Nevertheless, the prevalence of moderate or high FoF was high (66%), which may be explained by the fact that individuals who participate in a fall prevention project tend to be more sensitive to (fear of) falling. In addition, potential limitations by using the EQ-5D as measure of HrQoL should be noted. The EQ-5D excludes aspects of quality of life beyond health that may also be affected by fear of falling. Even some health-related aspects may not be sufficiently captured in the five dimensions of the EQ-5D. Although the introduction of the 5-level version of the EQ-5D has improved the ability to differentiate between health conditions, ceiling effects remain a problem [61, 62]. The results of this study should therefore be tested in future studies using different measures of (health-related) quality of life. Finally, the transferability of the results to other populations may be limited, because preference-based value sets for the German general population were used to calculate the EQ-5D index and country-specific cultural factors are known to influence the subjective assessment of health.

## Conclusion

FoF was a significant negative predictor of the EQ-5D index, whereas FoF did not predict HrQoL measured by the EQ-VAS. This is probably attributable to the different concepts underlying the EQ-5D index and the EQ-VAS. Furthermore, function and disability were shown to mediate the association between FoF and HrQoL. Therefore, future interventions should account for function and disability in their design.

## Data Availability

The datasets generated and/or analysed during the current study are not publicly available due to ethical and confidentiality concerns but are available from the corresponding author on reasonable request.

## Abbreviations

EQ-5D: EuroQoL-5D: an instrument for measuring health-related quality of life
EQ-5D-5L: 5-level version of the EQ-5D
EQ-VAS: Visual analogue scale of the EQ-5D
FES-I: Falls Efficacy Scale International
FoF: Fear of falling
HrQoL: Health-related quality of life
LiFE: Lifestyle-integrated functional exercise
LLFDI: Late life function and disability instrument
SD: Standard deviation
TUG: Timed up-and-go test
